# Effects of Christian Orthodox Fasting Versus Time-Restricted Eating on Plasma Irisin Concentrations Among Overweight Metabolically Healthy Individuals

**DOI:** 10.3390/nu13041071

**Published:** 2021-03-25

**Authors:** Spyridon N. Karras, Theocharis Koufakis, Lilian Adamidou, Georgios Dimakopoulos, Paraskevi Karalazou, Katerina Thisiadou, Kali Makedou, Kalliopi Kotsa

**Affiliations:** 1 First Department of Internal Medicine, Division of Endocrinology and Metabolism, Medical School, Aristotle University of Thessaloniki, AHEPA University Hospital, 54636 Thessaloniki, Greece; karraspiros@yahoo.gr (S.N.K.); thkoyfak@hotmail.com (T.K.); 2 Department of Dietetics and Nutrition, AHEPA University Hospital, 54636 Thessaloniki, Greece; loula1924@hotmail.com; 3 Medical Statistics, Epirus Science and Technology Park Campus of the University of Ioannina, 45500 Ioannina, Greece; info@biostats.gr; 4 Laboratory of Biological Chemistry, Medical School, Aristotle University of Thessaloniki, AHEPA University Hospital, 54636 Thessaloniki, Greece; vivikarala@gmail.com (P.K.); thisiadou@yahoo.gr (K.T.); kalimakedou@gmail.com (K.M.)

**Keywords:** Orthodox fasting, time-restricted eating, irisin, cardiometabolic health

## Abstract

Irisin has been recently identified as an adipomyokine produced during physical activity and involved in the browning of adipose tissue. Despite the emerging evidence suggesting an inverse relationship between irisin plasma concentrations and adverse metabolic outcomes, the exact impact of diet on irisin levels remains obscure. Thus, we aimed to assess the effects of two dietary patterns, Christian Orthodox fasting (OF) and 16:8 time-restricted eating (TRE), on circulating irisin levels among overweight, metabolically healthy, adults. Plasma irisin, glucose and lipid parameters, calcium homeostasis, and anthropometry were evaluated in 29 Orthodox fasters and 14 age and body mass index (BMI)-matched TRE controls (mean age and BMI, 48.8 years and 28.7 kg/m^2^, respectively) at three, distinct time points—before the implementation of the energy-restricted diets (baseline), at the end of the dietary intervention (7 weeks) and 5 weeks after participants returned to their typical dietary habits (12 weeks from baseline). Repeated measures analysis was applied to assess differences between the two groups and the effect of several indices on irisin levels at all three time points. At 12 weeks, the OF group manifested higher irisin concentrations compared with both its baseline values (64.3 ± 54.4 vs. 43.6 ± 42.2 ng/mL, *p* = 0.01) and those of the TRE group at the same time point (64.3 ± 54.4 vs. 44.2 ± 26.6 ng/mL, *p* = 0.04). Glycemic, lipid, and anthropometric parameters were not found to correlate with irisin levels. In contrast, parathyroid hormone (PTH) concentrations at 12 weeks correlated with irisin concentrations (*p* = 0.04), indicating that lower values of irisin are expected for higher PTH measurements. The findings of this pilot study suggest favorable long-term effects of OF on irisin levels. The interplay between irisin, PTH, and diet warrants further investigation.

## 1. Introduction

Fasting, as a means for the improvement of spiritual and body health, is gaining increasing popularity around the world with its effects, particularly on cardiometabolic health, having been put under the spotlight of medical research during the past few years. Christian Orthodox fasting (OF) is a variation of the typical Mediterranean Diet (MD), which is practiced by a significant part of the Orthodox population, for a total of 120 to 180 days during the calendar year [1]. Consistent with the Eastern Orthodox Church rules, fasters should avoid the consumption of meat, dairy products, and eggs daily, and abstain from fish and olive oil on specific weekdays during the fasting period. Previous research has demonstrated that OF is associated with a decrease in energy and fat and an increase in fiber intake [2,3] and results in an improved serum lipid profile [4]. In addition, Athonian monks have been shown to manifest more favorable metabolic profiles than Orthodox lay fasters, probably as a result of improved anthropometry, including low visceral fat (VF) mass, related to restricted energy intake during both fasting and non-fasting days and scheduled physical tasks [5].

Time-restricted eating (TRE) consists of a plan of intermittent fasting where food consumption is permitted only during a certain period of the day, and then fasters restrain from calorie-containing food for the rest of the day [6]. Although TRE does not directly involve limitations in energy intake, during the period of eating, it possibly leads to an overall calorie deficit [7]. Human and animal research has been so far indicative of a positive impact of TRE on systemic inflammatory status, serum adipokine concentrations, and human anthropometry, including body weight (BW) and fat mass [8]. We have recently demonstrated that OF and TRE promote comparable reductions in BW, with OF manifesting superior lipid-lowering actions than the TRE pattern and none of them exert significant effects on glycemic status [9,10]. 

Irisin was originally recognized as a myokine whose production is induced by exercise. The latter upregulates the expression of peroxisome proliferator-activated receptor (PPAR)-γ co-activator (PGC)-1α, leading to the expression of fibronectin type III domain containing (FNDC)5 [11]. Irisin is produced by FNDC5 cleavage and is implicated in the browning of adipose tissue [12]. More recent work identified the production of irisin from liver and adipose tissue as well [13], suggesting a potential role of this adipomyokine in mediating the complex interactions between exercise and metabolic health. However, a number of clinical studies have highlighted an inverse relationship between circulating irisin concentrations and adverse metabolic outcomes, including obesity, diabetes, and insulin resistance, and existing data are still inconclusive, highlighting the need for further evidence to support such a link [14]. Moreover, an inverse relationship between irisin and osteoporotic vertebral fractures among postmenopausal females, independently of fat, muscle mass (MM), bone mineral density and physical activity, has been previously described, suggesting potential favorable effects of irisin on bone quality [15,16], by promoting osteoblast differentiation [17]. There are very limited results, however, on the effects of diet on irisin concentrations, particularly with respect to OF and TRE patterns. 

This pilot study aimed to evaluate changes over time in irisin concentrations among overweight adults who followed two distinct nutritional advocacies—namely OF and TRE—for 7 weeks, and explore the potential effects of glycemic, lipid, calcium (Ca) homeostasis and anthropometric parameters on irisin levels.

## 2. Materials and Methods

### 2.1. Study Population

Participants in the study were staff of the AHEPA University Hospital and the Aristotle University of Thessaloniki, Greece, and were enrolled between December 2018 and February 2019. Individuals with Body Mass Index (BMI) >25 kg/m^2^ were considered eligible for inclusion. Specific exclusion criteria were the following—(i) chronic renal disease, severe liver disease, prediabetes/diabetes mellitus, arterial hypertension or uncontrolled hypothyroidism (ii) recent (within the past three months) surgical procedures or severe infections, (iii) treatment with drugs that affect BW, glucose metabolism, lipid profile and Ca or vitamin D status (e.g., statins, corticosteroids, antipsychotics), (iv) intake of vitamins or mineral supplements, (v) physical disabilities and/or neurodegenerative disorders leading to impaired physical mobility, (vi) acute infections and chronic degenerative diseases, (vii) amenorrhea for the past six months.

### 2.2. Dietary Intervention

The design and methods of dietary intervention used in the study have been previously reported [9,10]. In brief, the intervention lasted 7 weeks (48 days) and occurred during Lent fasting, preceding Orthodox Easter (March and April, 2019). Orthodox fasters and TRE participants followed an energy-restricted diet, providing a total of 1200–1500 kilocalories (kcal) (5020.8–6276 kilojoules (kJ)) per day for females and 1500–1800 kcal (6276–7531.2 kJ) per day for males. Participants’ total energy expenditure was estimated using the Mifflin–St Joer prediction equation to calculate basal metabolic rate and then multiplied by the individual’s activity level [18]. The result was adjusted for an expected weight loss of ≥0.5 kg (kg) per week (energy deficit of 500–750 kcal/2092–3138 kJ per week) and assessed according to the guidelines of the American Heart Association (AHA) for the management of overweight and obesity [19]. 

Orthodox fasters followed a diet incorporating the rules of Athonian fasting. In particular, they restricted animal products, except for two days during the fasting period, on which fish consumption was permitted. They were also advised to consume food exclusively from 08.00 to 20.00. TRE participants were requested to eat food from 08:00 to 16:00 h daily, and fast from 16:00 to 08:00 h daily. During the feeding window, participants were asked to conform with their dietary plans, which included two meals (08:00 and 13:00 h) and two snacks (11:00 and 15:30 h). During the fasting window, individuals were allowed to drink water or energy-free beverages. The TRE group was asked to avoid high-fat animal products such as whole dairy, full fat cheese, reformed meat, sweets high in animal fat, butter and whipped cream. They were advised to replace such food with low fat alternatives, such as low-fat dairy products (1.5–2%), poultry without skin, fish, lean beef or pork cuts. The TRE diet composition was 52–55% carbohydrates, 15–18% protein and 29–30% fat.

Amounts of each food group in OF and TRE groups were estimated after taking into consideration the principles of the Orthodox Church [3,5] and the Greek National Dietary Guidelines for Adults [20], respectively. Adherence to diets was evaluated with a 3-day food record (two weekday and one weekend day), using the Nutrition Analysis Software Food Processor (Food Processor Analysis Software 2018) [21]. Subjects were contacted at two time points during the study (at weeks 2 and 5), to check their adherence to the diet and see if any advice from the research team was needed. Following the end of the 7-week intervention, subjects were asked to return to their typical dietary habits. 

From baseline to week 12, both groups were strictly advised to keep a stable level of physical activity, namely, 150 min per week of moderate-intensity aerobic exercise (e.g., brisk walking or light effort bicycling) according to the AHA recommendations [19] and not to implement resistance training exercises.

### 2.3. Anthropometric Measurements

Anthropometric data were collected from study participants at three time points—baseline, 7 weeks from baseline (end of the dietary intervention), and 12 weeks from baseline. Height was measured with a Holtain wall stadiometer, whereas BW was recorded with the use of a calibrated computerized digital balance (K-Tron P1-SR, USA, Onrion IIc). BMI was calculated as the ratio of weight in kilograms divided by the height in meters squared (kg/m^2^). Body fat (BF), VF, MM and lean body mass (LBM), were assessed with the method of bioelectrical impedance analysis (BIA) (SC-330 S, Tanita Corporation, Tokyo). 

### 2.4. Biochemical Measurements

Blood samples were drawn early in the morning, following a 12-h overnight fast, by ante-cubital venepuncture and samples were kept at −20 °C prior to analysis. Ca, serum lipid (total cholesterol, (TC), high-density lipoprotein cholesterol (HDL-C), low-density lipoprotein cholesterol (LDL-C), triglycerides (TG)) and glycemic (fasting glucose and fasting insulin) profile determinations were performed using the system of automated analyzers COBAS 8000 (Roche Diagnostics GmbH, D-68298 Mannheim, Germany). Parathyroid hormone (PTH) and 25-hydroxy-vitamin D (25(OH)D) were assayed in a COBAS e 602 immunochemistry module using electrochemiluminescence (ECL) technology (Roche Diagnostics GmbH, D-68298 Mannheim, Germany).

The method of determination of serum levels of irisin was a competitive enzyme immunoassay. The immunoplate is pre-coated with a secondary antibody which can bind to the Fc fragment of the primary antibody. The Fab fragment of the primary antibody is then competitively bound by a biotinylated peptide and the targeted peptide in serum. The biotinylated peptide interacts with streptavidin–horseradish peroxidase which catalyzes the substrate solution. The intensity of the resulting color measured photometrically at 450 nm is inversely proportional to the concentration of the targeted peptide. The intra-assay variation is <0.01 and the inter-assay variation is <0.015. Results are expressed as ng/mL. Reference ranges of values other than that of irisin and inter-and intra-assay coefficients of variation for the examined parameters have been previously reported [9,10].

### 2.5. Data Management and Statistical Analysis

Standardized electronic case report forms (CRF) were used for data entry. Specific persons within the research team were responsible for data collection, CRF tracking, data entry and validation and discrepancy management, and an internal quality control procedure was applied to ensure the validity of the procedures.

Means and standard deviations were used to describe scale measurements of the study. Linear mixed models were applied to examine the effect of baseline measurements of BF, TG, LBM and PTH on irisin levels at all three time points, as well as the effect of the group. Sphericity was examined using Mauchly’s test and F tests were used to assess statistically significant factors and covariates. Pairwise comparisons that followed for each group across time were adjusted under the Bonferroni criterion. Post hoc power analysis showed a power exceeding 0.8 to detect differences between the three time points for the fasting group. Significance was set at 0.05 in all cases and the analysis was carried out with the use of the SPSS v.23.0.

### 2.6. Ethical Consideration

All procedures performed in the study conformed to the ethical standards of the Helsinki Declaration for medical research involving humans, as revised in 2008. Written informed consent was provided by each participant enrolled in the study. The research protocol was approved by the Ethics Committee of the AHEPA University Hospital (approval number 25224/2019).

## 3. Results

### 3.1. Study Population Characteristics and Changes in Anthropometry and Biochemical Parameters 

The study population comprised 43 participants (32 females, 74.4%). Among them, 29 were included in the OF group (22 females, 75.9%) and 14 in the TRE group (10 females, 71.4%). Mean age of the participants was 48.8 years, whereas all women enrolled were premenopausal. As presented in Table 1, the two groups did not differ in terms of baseline characteristics, including demographic, anthropometric, and biochemical features. All participants reported a high degree of adherence to the diets and there were no drop-outs.

Changes in anthropometry, lipid parameters, and glycemic indices throughout the study period in Orthodox fasters and TRE controls have been previously reported in detail [10]. In brief, both groups demonstrated reductions in BMI at the end of the intervention compared with baseline, with this pattern being consistent until the end of the follow-up period—OF: 29.0 ± 6.0 kg/m^2^ (baseline) vs. 28.2 ± 5.4 kg/m^2^ (7 weeks) vs. 27.9 ± 5.3 kg/m^2^ (12 weeks), *p* < 0.001 and TRE: 28.3 ± 6.7 kg/m^2^ (baseline) vs. 27.5 ± 6.3 kg/m^2^ (7 weeks) vs. 27.4 ± 6.4 kg/m^2^ (12) weeks, *p* < 0.001. The decrease in BW of individuals of the two groups was accompanied by a significant reduction in WC (OF: 92.4 ± 15.0 cm (baseline) vs. 91.9 ± 15.1 cm (7 weeks) vs. 91.1 ± 14.6 cm (12 weeks), *p* < 0.001 and TRE: 92.6 ± 16.4 cm (baseline) vs. 89.3 ± 16.0 cm (7 weeks) vs. 88.5 ± 15.6 cm (12 weeks), *p* < 0.001), but still not in BF or LBM.

In the OF group, lipid concentrations tended to decrease during fasting, increasing again at week 12 compared with their baseline values. This was applicable for TC (189 ± 36 mg/dL (baseline) vs. 174 ± 36 mg/dL (7 weeks) vs. 192 ± 42 mg/dL (12 weeks), *p* < 0.001), HDL-C (51 ± 11 mg/dL (baseline) vs. 49 ± 10 mg/dL (7 weeks) vs. 52 ± 11 mg/dL (12 weeks), *p* < 0.001) and LDL-C (117 ± 31 mg/dL (baseline) vs. 102 ± 30 mg/dL (7 weeks) vs. 120 ± 37 mg/dL (12 weeks), *p* = 0.001). In the TRE group, only HDL-C was found to be higher at week 12 compared to week 7 (62 ± 15 vs. 56 ± 14 mg/dL, *p* < 0.001). Neither of the groups manifested significant alterations in glycemic indices during the study period. 

### 3.2. Changes in Irisin Concentrations

Orthodox fasters presented increased irisin concentrations at weeks 7 and 12 compared with baseline; however, the difference was significant only between baseline and 12-week levels (*p* = 0.01). The estimated values were—43.6 ± 42.2 ng/mL (baseline) vs. 68.4 ± 51.5 ng/mL (7 weeks) vs. 64.3 ± 54.4 ng/mL (12 weeks). In the TRE group, irisin levels increased during the dietary intervention, following a decreasing trend between weeks 7 and 12. These differences were not significant between any time points—29.3 ± 27.2 ng/mL (baseline) vs. 65.6 ± 72.4 ng/mL (7 weeks) vs. 44.2 ± 26.6 ng/mL (12 weeks), *p* > 0.05. Moreover, intergroup comparisons of irisin concentrations yielded non-significant differences at baseline and at 7 weeks; nevertheless, a significantly higher mean value was observed at 12 weeks for the OF group (64.3 ± 54.4 vs. 44.2 ± 26.6 ng/mL, *p* = 0.04). Table 2 presents the fluctuations of irisin values in the two groups throughout the study period. 

### 3.3. Effects of Various Parameters on Irisin Levels

**Figure 1:**
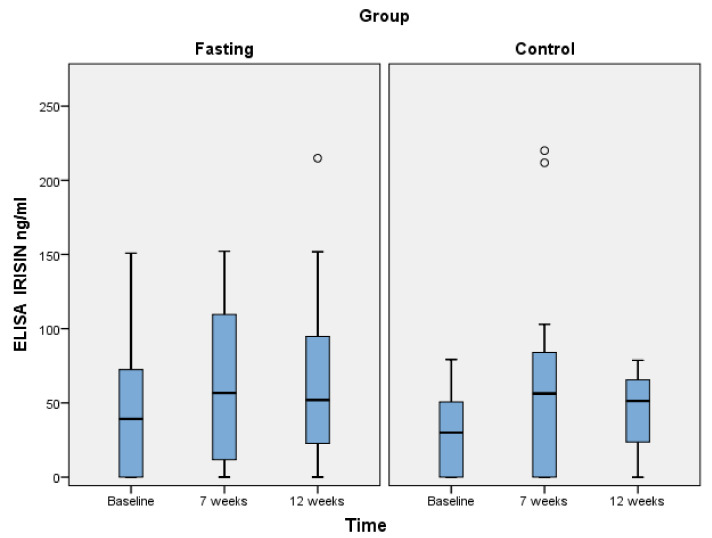
Differences between parathyroid hormone and irisin concentrations in the entire study population at baseline, 7, and 12 weeks. Abbreviations: Fasting: Orthodox fasting group; Control: Time-restricted eating group.

Among the variables examined for their effect on irisin values and for the entire study population, borderline non-significant correlations (*p* = 0.06) were detected for lipids. Specifically, for TC, TG, HDL-C and LDL-C concentrations, the obtained *p*-values were 0.06, 0.08, 0.06 and 0.06, respectively. Of those, only the correlation between irisin and TC was positive, indicating that larger values of irisin would be expected for larger values of TC, whereas TG, HDL-C and LDL-C would be reversely associated. The only statistically significant effect was detected at 12 weeks (*p* = 0.04) for PTH, indicating that lower values of irisin are expected for higher PTH measurements. This was not statistically significant at baseline (*p* = 0.09) nor at 7 weeks (*p* = 0.53). Figure 1 presents the evolution of irisin concentrations throughout the study period in the two groups.

The interaction term between PTH and the group was also not statistically significant at any time point, indicating that these relationships are observed in both the OF and the TRE group. The *p*-values for baseline, 7 weeks, and 12 weeks were 0.61, 0.71, and 0.99, respectively, although these relatively high *p*-values may also be due to lack of power regarding the true effect of the interaction term. Worth noting is that the observed relationship was not affected by 25(OH)D levels at any time. Similarly, 25(OH)D levels did not seem to affect the relationships between irisin and LBM, irisin and total BF, or irisin and VF, which were anyhow non-significant at all time points.

## 4. Discussion

In this prospective study, overweight, metabolically healthy adults were asked to follow two energy-restricted diets for 7 weeks, and subsequently returned to their standard eating habits for another 5 weeks. It was demonstrated that Orthodox fasters manifested higher irisin concentrations at the end of the follow-up period compared with both their baseline values and the levels of the TRE group at the same time point. In addition, lipid and glycemic parameters were not found to correlate with irisin levels. In contrast, we observed an inverse association between PTH and irisin values at 12 weeks. To the best of our knowledge, this is the first study to assess changes in irisin concentrations in individuals following either an OF or a TRE pattern.

Despite the crucial role that diet plays in affecting metabolic risk factors and the link between irisin and metabolic health, previous studies have produced contradictory results regarding the potential of dietetic interventions to modulate circulating irisin levels. An older study failed to prove an association between food intake and irisin [22]. Subsequent research demonstrated that irisin levels are positively correlated with fruit consumption and negatively with meat eating [23], whereas others showed that increased energy intake results in a reduction in irisin levels [24]. In one study including individuals practicing Ramadan fasting—a type of religious fasting which involves no eating and drinking from sunrise to sunset—Alzoughool et al. demonstrated decreased irisin profiles after fasting compared with pre-fasting values [25]. In a cohort of individuals with metabolic syndrome who underwent an energy-restricted program, weight loss was accompanied by a depletion in irisin levels, which correlated with the reduction in atherogenic-related lipid parameters, independently of changes in BW [26]. Osella et al. [27] evaluated the effects of three different diets, namely, Low Glycemic Index (LGID), classical MD, and Low Glycemic Index MD, on irisin serum levels in individuals with metabolic syndrome. Mean irisin values increased in all diet groups, but only the LGID group reached statistical significance. It is worth noting that the interaction became significant at the sixth month of evaluation, implying that the effects of diet on irisin concentrations might take time to be evident. 

Previous results indicated that exercise interventions result in elevated irisin levels [28]. Morelli et al. demonstrated higher serum irisin concentrations in individuals who performed vigorous-intensity physical activity, compared with those being physically inactive, despite having similar adherence to MD [29]. High-intensity exercise has been related to greater irisin response than low-intensity exercise under comparable energy consumption [30]. Moreover, irisin levels were significantly increased following an 8-week resistance training program but remained unaffected by aerobic training [31], suggesting that the intensity and type of physical activity interfere with irisin concentrations. Taken together, the above-mentioned observations might offer a reasonable explanation for the reason that moderate-intensity aerobic exercise followed by the participants of this study did not have a significant impact on the irisin levels of the TRE group. 

There is growing evidence pointing towards positive effects of TRE plans on cardiometabolic health markers, including adipokine profiles. A very recent study demonstrated significant increases in adiponectin levels following a 28-day TRE protocol among physical active, still overweight (BMI 28.5 ± 8.3 kg/m^2^), college-age men, with this benefit being independent of calorie intake and probably deriving from the time restriction character of this nutritional advocacy per se [32]. However, the role of TRE in modulating circulating irisin concentrations has not been so far investigated. Interestingly, irisin secretion has been shown to follow a day–night rhythm, with a peak at 9:00 p.m. [22], whereas other studies are indicative of possible differences in irisin release dependent on the time of the day when exercise is performed [33]. Thus, the chronobiological interplay between nutrition, physical activity, and irisin deserves further evaluation in future studies. 

Our observation of an inverse association between PTH and irisin concentrations at 12 weeks in the entire study population has been consistently reported, indicating a reverse musculoskeletal biological interaction. In detail, in vitro data suggest a negative regulatory effect of both short-term (3 h) and long-term (6 days) treatment with PTH on FNDC5mRNA and protein expression in myotubes, by acting through the PTH receptor, which subsequently activates Erk1/2 phosphorylation, whereas plasma irisin has been shown to be lower among post-menopausal females with primary hyperparathyroidism than controls [34]. In addition, irisin presents an inverse association with PTH in postmenopausal women who have low bone mass [15]. These results imply that irisin downregulates the expression of the PTH receptor in osteoblasts, indicating an inhibitory action on PTH effects on osteoblast formation, apart from inducing osteoblast function and expression. In the context of our findings, it could be postulated that additional parameters, including dietary ones, might be implicated in the intriguing association between irisin and bone metabolism.

The results of our study should be considered in the light of its limitations. The small sample size and the short period of the intervention might have limited its power to reveal further changes in the investigated parameters. The method of bioelectrical impedance analysis used for body composition assessment presents specific limitations, given that the measurements can be affected by clinical status, particularly the presence of edema [35]. The latter, along with the short period during which participants followed the diet, could explain the absence of changes in BF despite the significant reductions in BW. An additional limitation is that randomization has not been applied to the formation of the two study groups. This is because practicing religious fasting is closely related to the religious beliefs of the participants and randomization would not be feasible without violating these beliefs. The impact of physical activity on the results and its interaction with the diets and irisin levels should be considered; however, the fact that participants were requested to follow a standard pattern of exercise during the study period might have attenuated the confounding effects of this parameter. 

An interesting finding of the present study is that Orthodox fasters continued to improve their irisin status, even after the end of fasting and up to the end of the follow-up period, despite adopting their standard eating habits after week 7. Previous research has associated the adoption of OF with improved life satisfaction and wellbeing [36] and decreased prevalence of depression and anxiety [37]. Thus, we speculate that for the above-mentioned reasons, in conjunction with their strong religious beliefs, some of the participants in the OF group preferred not to entirely abandon OF and incorporated elements of this diet into their habitual nutritional plans even during the non-fasting period. Moreover, the sex ratio in the study does not reflect the sex distribution of the Greek population. On the other hand, it should be considered that from a strictly cultural and religious perspective, lay women are more consistent with fasting compared with lay men [38] and thus, the overrepresentation of women in the study population is not unexpected. Sex-specific biological responses to OF remain an area for future studies. Finally, under-reporting of energy intake has been shown to be a key limitation of self-reported dietary intake [39] and this should be seen as an additional limitation of the present study. 

## 5. Conclusions

In conclusion, the results of our study suggest that the adoption of OF might have favorable long-term effects on irisin concentrations. However, the association between diet and irisin is mediated by numerous parameters, including patient selection, differences in diet composition, physical activity, adherence, and study duration, which might prove critical factors in determining variability in the results among different studies. Given the pilot character of this study, our findings need to be replicated by trials with larger sample sizes and longer duration to draw definite conclusions. 

## Figures and Tables

**Table 1:** Baseline features of the two groups.

Parameter	TRE Group	OF Group	*p*-Value
Participants; Women [*n* (%)]	14; (71.4%)	29; (75.9%)	0.75
Age (years)	46.3 ± 8.7	49.9 ± 8.9	0.21
Weight (kg)	77.4 ± 20.2	77.6 ± 17.1	0.97
BMI (kg/m^2^)	28.3 ± 6.7	29.0 ± 6.0	0.75
Waist circumference (cm)	92.6 ± 16.4	92.4 ± 15.0	0.98
Body fat (%)	32.5 ± 7.3	35.4 ± 9.1	0.29
Lean body mass (kg)	51.6 ± 12.7	47.5 ± 9.9	0.26
TC (mg/dL)	191.0 ± 25.0	189.0 ± 36.0	0.81
HDL-C (mg/dL)	58.8 ± 17.8	51.2 ± 11.1	0.09
HDL-C (mg/dL)	117.0 ± 19.6	117.0 ± 30.9	0.99
TG (mg/dL)	78.2 ± 21.1	103.4 ± 46.8	0.63
FPG (mg/dL)	90.1 ± 11.7	83.0 ± 8.5	0.07
FPI (μIU/mL)	19.4 ± 28.2	10.6 ± 8.4	0.27

Data are presented as mean ± standard deviation Abbreviations: OF: Orthodox fasting; TRE: time restricted eating; BMI: Body Mass Index; TC: Total cholesterol; HDL-C: high-density lipoprotein cholesterol; LDL-C: low-density lipoprotein cholesterol (LDL-C); TG: triglycerides; FPG: Fasting plasma glucose; FPI: Fasting plasma insulin.

**Table 2:** Evolution of irisin concentrations throughout the study period in the two groups.

Irisin (ng/mL)					*p*-Value
Group		Baseline	7 Weeks	12 Weeks	
OF (*n* = 29)	Mean (SD)	43.6 (42.2)	68.4 (51.5)	64.3 (54.4)	Baseline vs. 7 w: 0.21 Baseline vs. 12 w: **0.01** 7 w vs. 12 w: 0.65
TRE (*n* = 14)	Mean (SD)	29.3 (27.2)	65.6 (72.4)	44.2 (26.6)	Baseline vs. 7 w: 0.10 Baseline vs. 12 w: 0.73 7 w vs. 12 w: 0.24
*p*-value		0.28	0.99	**0.04**	

Abbreviations: OF: Orthodox fasting; TRE: time restricted eating; SD: standard deviation; w: week. Values in bold indicate significant differences.

## Data Availability

The data presented in the study are available on request from the corresponding author.
